# T cell-prolymphocytic leukemia detected in a patient of breast cancer at the time of recurrence: a case report

**DOI:** 10.1186/1757-1626-3-4

**Published:** 2010-01-04

**Authors:** Manish Singhal, Vinod Raina, Ritu Gupta, Prasenjit Das

**Affiliations:** 1 Department of Medical Oncology, Institute Rotary Cancer Hospital (IRCH), All India Institute of Medical Sciences (AIIMS), New Delhi, India; 2 Department of Lab Oncology, IRCH, AIIMS, New Delhi, India; 3 Department of Pathology, AIIMS, New Delhi, India

## Abstract

**Introduction:**

Therapy related second malignancy of the hematological system is small but real risk after adjuvant chemotherapy for breast cancer. It includes acute myeloid leukemia (AML) and myelodysplastic syndrome (MDS); however T-cell prolymphocytic leukemia (T-PLL) has not been described earlier in relation to breast cancer and its therapy. T-PLL is a rare chronic T-cell lymphoproliferative disease with a mature post-thymic T-cell immunophenotype and aggressive clinical course.

**Case presentation:**

A 45 year old Indian female of Nordic origin presented 5 years back with a lump in the right breast and the axilla. She underwent modified radical mastectomy. Histophotomicrograph of the excised breast lesion showed a 2.1 cm duct carcinoma, positive for ER and PR with 1 out of 25 lymph nodes positive for metastasis. She received 6 cycles of chemotherapy with cyclophosphamide, epirubicin, and 5-fluorouracil. This was followed by tamoxifen 20 mg per day for five years. She was doing well on follow up until the completion of fifth year of her disease, when she presented with complaints of mild fever and weakness. Examination revealed generalized lymph node enlargement along with hepatomegaly.

Hemogram showed mild anemia, normal platelet count and a leukocyte count of 1.2 × 10^11^/L. Peripheral blood examination revealed medium sized lymphoid cells, constituting almost 75% of total nucleated cell population. Immunophenotying, established a diagnosis of post thymic T-cell prolymphocytic leukemia. Contrast-enhanced computed tomography of the chest and abdomen was done which revealed an anterior mediastinal mass with destruction of sternum along with multiple small nodular shadows in bilateral lung fields suggestive of lung metastasis. Fine needle aspiration cytology of the mass showed atypical ductal cells with nuclear pleomorphism, which were positive for ER, PR and Her2neu protein. This confirmed a co-existent metastatic breast carcinoma. She was started on chemotherapy for T-PLL along with hormonal therapy with aromatase inhibitor. Unfortunately, both her malignancies progressed after an initial stable disease of two months.

**Conclusion:**

Our case describes the potential of breast chemotherapy to cause grave second hematological malignancies of the T-cell lymphoid lineage, not described earlier. Such events highlight the importance to identify those patients of breast cancer in whom chemotherapy can safely be avoided.

## Introduction

Therapy related second malignancy of the hematological system is small but real risk after adjuvant chemotherapy for breast cancer. It includes acute myeloid leukemia (AML) and myelodysplastic syndrome (MDS); however T-cell prolymphocytic leukemia (T-PLL) has not been described earlier in relation to breast cancer and its therapy. T-PLL is a rare chronic T-cell lymphoproliferative disease with a mature post-thymic T-cell immunophenotype and aggressive clinical course.

## Case Presentation

**Figure 1:**
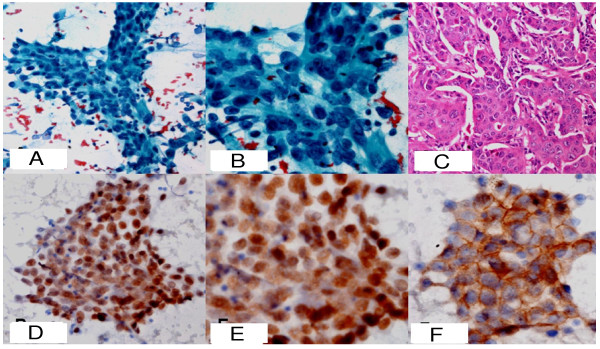
**A. Low power photomicrograph of the FNA smear from anterior mediastinal mass showing atypical ductal cells arranged in trabecular pattern (PAP ×100)**. **B**. The same ductal cells showing moderate nuclear pleomorphism, overlapping and prominent nucleoli (PAP × 400). **C**. Histophotomicrograph of the excised primary breast lesion showing malignant ductal cells arranged in trabeculae (H&E × 200). **D**. Ductal cells form the FNA of anterior mediastinal mass showing nuclear positivity for ER protein (IHC-ER×100). **E**. Same ductal cells showing positivity for PR protein (IHC-PR × 200). **F**. Same ductal cells showing grade 3 positivity for erb-B2 (Her2neu) stain (IHC-ERBB2 × 200).

**Figure 2:**
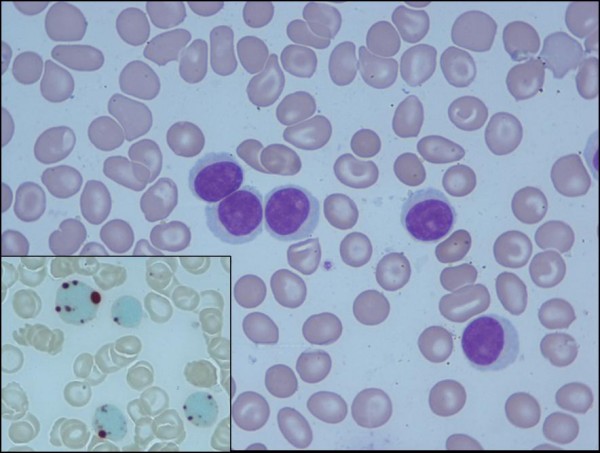
**Peripheral blood smear showing medium sized lymphoid cells, with oval nucleus, a visible nucleolus and moderate amount of basophilic agranular cytoplasm (Jenner-Giemsa, 1000×)**. **Inset **Lymphoid cells showing, dot-like staining with acid phosphatase, 1000×).

A-45-year old Indian female of Nordic origin presented 5 years back with a lump in the right breast and the axilla. She was detected to have carcinoma of the right breast with clinical stage as T2N1. Investigations ruled out any metastatic site and she underwent modified radical mastectomy. The tumor measured 2.1 cm with 1 out of 25 lymph nodes positive for tumor deposits. Histophotomicrograph of the excised breast lesion showed a duct carcinoma, not otherwise specified. (Figure. 1C). Immunohistochemistry showed positivity for estrogen receptor(ER) and progesterone receptor (PR). She received 6 cycles of chemotherapy with cyclophosphamide (500 mg/m2), epirubicin (50 mg/m2), and 5-fluorouracil (500 mg/m2) on day1 and day15 every 4 weeks. This was followed by tamoxifen 20 mg per day for five years. She was doing well on follow up until the completion of fifth year of her disease, when she presented with complaints of mild fever, weakness and swelling in the neck and elbow. Examination revealed bilateral cervical, epitrochlear, inguinal and right axillary lymph node enlargement along with hepatomegaly. Hemogram showed mild anemia, normal platelet count and a leukocyte count of 1.2 × 10^11^/L. Lactate dehydrogenase level was elevated (746 IU/L). Peripheral blood examination revealed medium sized lymphoid cells (Figure 2), constituting almost 75% of total nucleated cell population, suggestive of a chronic lymphoproliferative disorder. Findings were confirmed on bone marrow examination. The lymphoid cells showed, dot-like staining with acid phosphatase (Figure 2. inset) and immunophenotying, depicted positivity for CD2, CD3, CD4, CD5, CD7, CD45, CD38 and ZAP70 and negative for CD8, CD10, CD19, CD20, CD103, CD11c, CD23, surface immunoglobulin and TdT. Based on these findings, a diagnosis of post thymic T-cell prolymphocytic leukemia was made. Conventional cytogenetics did not reveal any abnormality.

**Figure 3:**
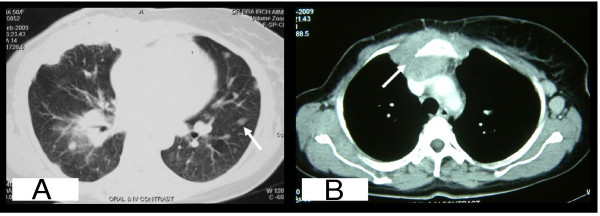
**A. CECT of chest showing anterior mediastinal mass with destruction of sternum and right coastal margin**. **B**. CECT of chest showing multiple small nodular shadows in bilateral lung fields.

Contrast-enhanced computed tomography (CECT) of the chest and abdomen was done which revealed an anterior mediastinal mass with destruction of sternum and right coastal margin (Figure. 3A arrow) along with multiple small nodular shadows in bilateral lung fields suggestive of lung metastasis (Figure. 3B arrow). In the background of breast carcinoma, recurrence of the primary breast cancer was kept as a differential and fine needle aspiration (FNA) cytology of the anterior mediastinal mass was done. Low power photomicrograph of the FNA smear showed atypical ductal cells arranged in trabecular pattern (Figure. 1A). The ductal cells showed moderate nuclear pleomorphism, overlapping and prominent nucleoli (Figure. 1B). Immunohistochemical stains performed on aspiration smears showed nuclear positivity for ER protein (Figure. 1D), PR protein (Figure. 1E) and grade 3 positivity for erb-B2 (Her2neu) stain (Figure. 1F). This confirmed a metastatic carcinoma consistent with a breast primary. She was started on chemotherapy for T-PLL with fludarabine and cyclophosphamide along with hormonal therapy with aromatase inhibitor (letrozole 2.5 mg per day) for metastatic carcinoma of the breast. Unfortunately, both her malignancies progressed after an initial stable disease of two months.

## Discussion

Leukemia is not an important factor in the natural history of breast cancer, though its association with breast cancer therapy has frequently been reported. There are reports of therapy related hematological malignancies in breast cancer dated as early as 1980's describing the incidence as 1.68 ± .33% at 10 years with chemotherapy vis a vis 0.06% to 0.27% with surgery alone [1]. In 1992, a large case-control study of 82,700 women with breast cancer demonstrated a relative risk of developing AML as 2.4 for radiotherapy, 10 for therapy with alkylating agents and 17.4 when the two were used in combination [2] The chemotherapy regimens employed during these studies used mainly melphalan as alkylating agent and that for long durations (12 to 24 months) [1,2]. Though over the last decade anthracyclines along with safer alkylating agents have formed the main core of the breast cancer therapy; the modern chemotherapy is not entirely safe. The more recent reports in the background of anthracycline and alkylator based chemotherapy have described the risk as, 0.5% estimated leukemia rate at 10 years with relative higher risk for more intense regimens (RR, 6.16; P < 0.0001) [3,4]. Studies have also remarked on a dose risk relation in respect to exposure to anthracycline as well as alkylators; the risk increasing with increasing cumulative doses [5].

Interestingly, the reports on second hematological malignancies after treatment for breast cancer mostly describe myeloid malignancies/MDS in contrast to lymphoid malignancies [1,5,6]. While therapy related lymphoid malignancies are relatively rare, T-PLL has never been listed as a possible therapy related second malignancy after treatment for breast cancer. This may have several attributes such as more pronounced impact of cytotoxic therapy in respect to telomere length dynamics and its accelerated shortening observed in myeloid lineage as opposed to lymphoid lineage before the development of MDS/AML after hematopoietic stem cell transplant [7]. However, the risk of therapy related lymphoid malignancies is not deniable. T-PLL is a disease of the elderly with median age 65-70 years and our patient was much younger to be at a natural risk for this disease [8]. Absence of family history of breast cancer and other malignancies, undermined the possibility of a common genetic abnormality or pathway for such an occurrence [9]. Also the disease appeared at 5 years after the completion of therapy which is the median latency time described for therapy related second malignancies of the hematopoietic system [1,3]. She also seemed to be at a relatively higher risk, being exposed to higher cumulative doses of anthracycline (600 mg/m2 of epirubicin) and alkylating agent (6 gm/m2 of cyclophosphamide) [5]. Our patient was discovered to have disease recurrence simultaneously with the detection of leukemia. Similar situation has also been reported by Fisher et al wherein 2 of 34 patients with therapy related hematological malignancy had simultaneously developed disease recurrence and myeloproliferative disorder [1]. This however, largely seems to be just a coincidence than otherwise.

T-cell PLL is an aggressive leukemia with median survival of 7-8 months [8,10]. Nucleoside analogues have been used with overall and complete response rates of 50% and 10% respectively [11]. Alemtuzumab which is a humanized IgG1 antibody that targets CD52, expressed at high density on the malignant T-cells, has been shown to be particularly effective with overall and complete response rates of 76% and 60% respectively [10]. However, responses are transient and further disease progression inevitable and the only possibility of cure lies in an allogeneic stem cell transplant [8]. Our patient was initially asymptomatic from the recurrence of her primary disease and was hence, prescribed hormonal therapy (letrozole) alone for breast carcinoma, along with fludarabine based chemotherapy for T-PLL. However, she progressed early in regard to both her malignancies, and this posed a major therapeutic challenge.

## Conclusion

Our case describes the potential of breast cancer chemotherapy to cause grave second hematological malignancies of the T-cell lymphoid lineage, not described earlier; and underscores the therapeutic dilemma posed by the co-existence of two aggressive malignancies. While breast cancer chemotherapy improves survival, events like this, reminds us of the importance to identify those who are at low-risk and in whom breast cancer chemotherapy can safely be avoided.

## Abbreviations

AML: Acute myeloid leukemia; MDS: Myelodysplastic syndrome; T-PLL: T-cell prolymphocytic leukemia; IU: International unit; ER: Estrogen receptor; PR: Progestrone receptor; T: Tumor stage; N: Nodal stage; CD: Cluster differentiation; ZAP: Zeta-associated protein; TdT: Terminal transferase detection; FNA: Fine needle aspiration; CECT: Contrast enhanced computed tomography; RR: Relative risk.

## Consent

Written informed consent was obtained from the patient for publication of this case report and accompanying images. A copy of the written consent is available for review by the Editor-in-Chief of this journal.

## Competing interests

The authors declare that they have no competing interests.

## Authors' contributions

MS was involved in immediate care of the patient, conducting planned investigations, and writing of the manuscript. VR was involved in concept and final approval of the manuscript. RG and PD were involved in establishing diagnosis, planning investigations and RG also helped with the manuscript. All authors read and approved the final manuscript.
